# Navigating the complexity of skull base osteomyelitis: a case study and comprehensive review

**DOI:** 10.1093/jscr/rjae282

**Published:** 2024-05-02

**Authors:** Kenza Horache, Manal Jidal, Najwa El Kettani, Meriem Fikri, Mohamed Jiddane, Firdaous Touarsa

**Affiliations:** Neuroradiology Department, Specialty Hospital of Rabat, Rue Lamfadel Cherkaoui Rabat - Institut B.P 6527, Morocco; Neuroradiology Department, Specialty Hospital of Rabat, Rue Lamfadel Cherkaoui Rabat - Institut B.P 6527, Morocco; Neuroradiology Department, Specialty Hospital of Rabat, Rue Lamfadel Cherkaoui Rabat - Institut B.P 6527, Morocco; Neuroradiology Department, Specialty Hospital of Rabat, Rue Lamfadel Cherkaoui Rabat - Institut B.P 6527, Morocco; Neuroradiology Department, Specialty Hospital of Rabat, Rue Lamfadel Cherkaoui Rabat - Institut B.P 6527, Morocco; Neuroradiology Department, Specialty Hospital of Rabat, Rue Lamfadel Cherkaoui Rabat - Institut B.P 6527, Morocco

**Keywords:** skull base osteomyelitis, intra clival abscess, MRI

## Abstract

Skull base osteomyelitis is a rare and life-threatening infection of the skull base, commonly seen in elderly diabetic patients as a result of otogenic or paranasal infection. The diagnosis is based on a series of arguments, including a high clinical suspicion, imaging findings, negative biopsies for malignancy, and microbiological isolation. Complications, including abscess formation and vascular involvement, mandate a multidisciplinary treatment approach, primarily involving broad-spectrum antibiotics and surgical debridement, but the prognosis is usually poor. Herein, we describe the case of a 55-year-old male, who 15 years prior, underwent radiation therapy for nasopharyngeal carcinoma. He presented with an infection of the skull base with extensive bone erosion accompanied by an uncommon complication; an intra clival abscess. Despite aggressive antibiotic therapy, the patient ultimately succumbed to septic shock.

## Introduction

Skull base osteomyelitis (SBO) is a rare but clinically significant infection, usually affecting elderly diabetic patients following otogenic or paranasal infections [1]. Despite its rarity, SBO poses significant diagnostic and therapeutic challenges in clinical practice, necessitating a multidisciplinary approach based on clinical evidence, imaging, negative biopsies for malignancy, and microbiological isolation [1], and it is predominantly caused by *Pseudomonas aeruginosa*, followed by *Staphylococcus aureus* [1, 2].

Moreover, treatment for SBO demands a concerted multidisciplinary approach, typically involving aggressive antibiotic regimens, surgical interventions for debridement, and meticulous management of associated complications.

In this article, we explore the complexities of SBO through a detailed case study and review of pertinent literature. We discuss the diagnostic challenges, treatment options, and potential complications associated with this condition.

## Case presentation

A 55-year-old man with a medical history of nasopharyngeal squamous cell carcinoma underwent 15 years prior treatment involving concurrent chemoradiotherapy.

Subsequent surveillance until 2015 yielded no evidence of nasopharyngeal carcinoma (NPC) recurrence. In 2017, the patient was diagnosed with diabetes mellitus, managed with oral antidiabetics, and additionally presented a history of recurrent right otitis media.

The patient presented to the emergency department with complaints of headaches, right facial pain, hearing loss, dysphagia, and diplopia. Two months prior to the admission, he experienced otalgia and right ear discharge.

Upon physical examination, the patient exhibited pyrexia, auricular swelling, perichondritis, and periauricular cellulitis. Neurological evaluation revealed cranial nerve involvement, affecting the right sixth and fifth cranial nerves.

Laboratory findings demonstrated leukocytosis (15.000 cells/mm^3^) and elevated level of C-reactive protein (120 mg/L).

**Figure 1:**
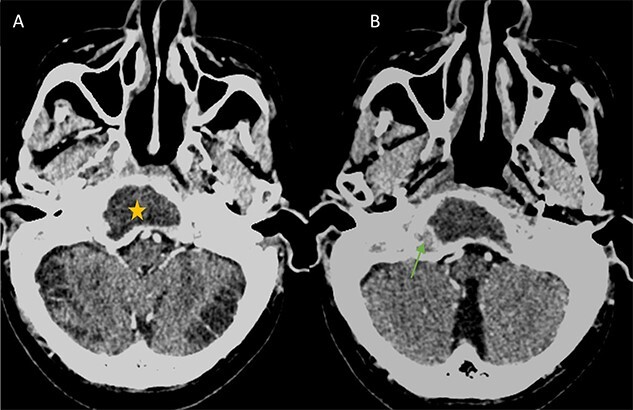
(A) Axial CT in soft-tissue window showing an intra clival fluid collection (star). (B) Axial Post contrast CT showing a peripheral enhancement (arrow).

**Figure 2:**
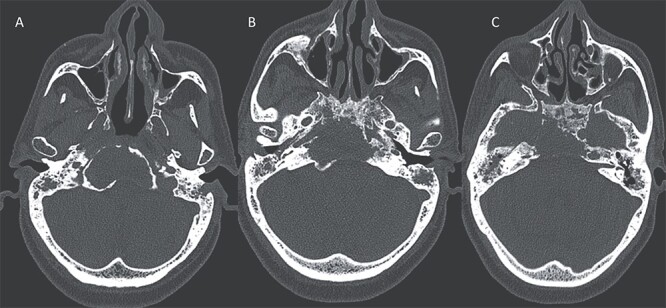
Axial CT in bony window showing: (A, B) extensive bone erosion of the clivus and the body and right greater wing of the sphenoid. (C) Soft tissue in the middle ear cavity and mastoid cells.

A computed tomography (CT) scan with intravenous contrast was performed (Figs 1 and 2), it revealed:

- A 3 × 2.5 × 3.2 cm low-attenuating intra clival mass with peripheral enhancing, accompanied by extensive bone erosion of the clivus.
- Bone destruction extending to the petrous, tympanic, and mastoid portions of the temporal bone, with involvement of the body and right greater wing of the sphenoid as well as the occipital bone, and concomitant sequestrum formations within the necrotic bones.
- Presence of soft tissue in the middle ear cavity and in the mastoid cells.

**Figure 3:**
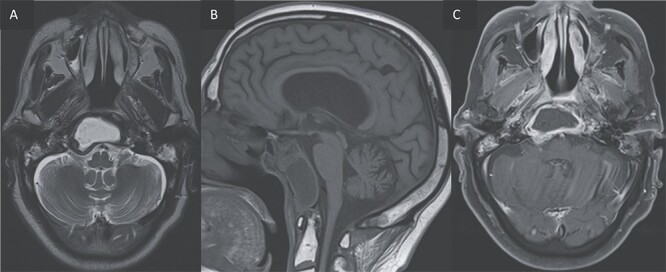
(A, B) Axial T2 and sagittal T1 images showing an expansile cystic lesion in the center of the clivus hyperintense in T2, hypointense in T1, with a rim hypointense in T2-weighted image. (C) Post-gadolinium T1-weighted shows a peripheral rim enhancement of the clival lesion.

**Figure 4:**
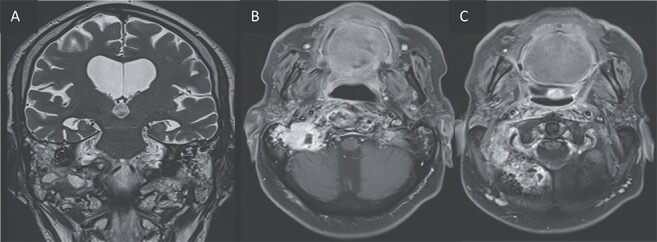
T2 coronal (A) and T1 post gadolinium axial images (B, C) showing the extension of the inflammation around the clivus and in the petrous, tympanic, and mastoid portions of the right temporal bone with small abscesses along the mastoid cells and the occipital bone.

For better diagnostic assessment, the patient underwent a magnetic resonance imaging (MRI) (Figs 3 and 4), it showed the following findings:

- An expansile cystic-necrotic lesion localized in the central region of the clivus, characterized by a hypointense rim on T2-weighted images.
- Post-gadolinium T1-weighted imaging delineating the cystic center with thick peripheral rim enhancement.
- Diffusion-weighted imaging (DWI) and apparent diffusion coefficient (ADC) maps demonstrating central restricted diffusion.
- Furthermore, inflammatory changes were noted to extend into the petrous, tympanic, and mastoid segments of the right temporal bone, as well as involving the petrous and mastoid regions of the left temporal bone and the occipital bone. Additionally, small abscess cavities were observed within the right occipital condyle.

Following an endoscopic transsphenoidal biopsy, purulent materiel was obtained. Anatomopathological analysis revealed subacute inflammatory changes, with no evidence of malignancy. Microbiological cultures yielded growth of *P. aeruginosa*.

According to the high clinical suspicion (diabetic patient with a history of radiation and recurrent otitis media), the radiological findings and the microbiological isolation, the diagnosis of bacterial SBO complicated by an intra clival abscess has been established.

The patient was administered an IV high dose of broad-spectrum antibiotics with control of the diabetic status. Unfortunately, the patient did not survive due to septic shock.

## Discussion

SBO stands as a rare yet severe infection affecting the temporal, sphenoid, or occipital bones. Predominantly originating from otogenic or sinonasal sources [1], SBO presents diagnostic challenges, occasionally mimicking malignancies on imaging studies.

Predisposing factors for SBO predominantly encompass elderly patients with diabetes or compromised immune systems [1–3]. Indeed, elevated pH levels in cerumen among diabetic patients create a better environment for bacterial growth. Additionally, diabetes can lead to microangiopathic changes that facilitate infection dissemination [1, 2].

Furthermore, a history of radiation therapy poses an additional risk, impairing bone vascularity and oxygen delivery, thereby predisposing patients to SBO [1, 2].

Notably, this condition may manifest months or years post-radiotherapy, with increased incidence correlating with higher radiation doses and prolonged treatment courses [4].

Other risk factors, including malignancies, small vessel disease, osteoporosis, Paget’s disease, and malnutrition, have been described [1–3].

SBO typically arises from inadequately treated chronic infections [1, 2]:

- Otogenic infections: often involve patients with diabetes and recurrent otitis externa, chronic suppurative otitis media, or mastoiditis.
- Non-otogenic infections: may originate from distant infections such as chronic sinusitis, odontogenic infections, or scalp infections.

Clinical manifestations of SBO encompass diverse symptoms, including headaches, facial pain, otalgia, purulent ear discharge, hearing loss, and cranial nerve palsies. Cranial nerve involvement is a common complication, often resulting in facial paralysis, hearing impairment, and diplopia.

Imaging investigations play pivotal roles in SBO evaluation, but it is often nonspecific.

CT imaging is essential for bone evaluation. It reveals bone erosion, demineralization, and bony sequestra within or surrounding necrotic bones [1].

Moreover, contrast-enhanced CT reveals soft-tissue infiltration, obliteration of fat planes, potential involvement of skull base foramina, and identification of vascular complications [1].

MRI helps delineating the extent of the infection. It visualizes soft-tissue enhancement, infiltration and enhancement of bone marrow, soft-tissue edema, obliteration of fat planes, dural enhancement, intracranial and perineural extensions, and potential involvement of adjacent anatomical structures [1, 2]. Typically, restricted diffusion is commonly observed on DWI. Notably, ADC values in patients with SBO tend to surpass those observed in individuals with malignant conditions [1].

The histopathological examination assumes critical importance, given the diagnostic challenge of distinguishing between tumor and SBO. Typically, findings reveal evidence of chronic inflammation and osteomyelitic bony changes [1, 2].

Causative pathogens predominantly include *P. aeruginosa* and *S. aureus*, with less common involvement of *Staphylococcus epidermidis*, *Salmonella*, *Proteus mirabilis*, and *Mycobacterium* species [1, 3].

Fungal etiologies, namely *Aspergillus* and *Candida* species, are also occasionally implicated.

According to Alvarez *et al.*, the diagnosis of SBO should be based on four points [1]:

- High index of clinical suspicion,
- Radiological evidence of infection,
- Repeated biopsies to rule out malignancy,
- Positive microbiologic isolation.

If not properly handled, multiple complications can occur [1]:

- Abscesses: discernible by areas of liquid signal intensity with restricted diffusion and ring enhancement. The individualization of abscess is an additional argument for SBO.
- Phlegmon: characterized by soft-tissue enhancement.
- Vascular complications: internal carotid artery bleeding, venous sinus thrombosis, arterial pseudoaneurysms, and ischemic infarction.
- Other infectious complications: meningitis, encephalitis, or intracranial abscesses.

Differential diagnosis of SBO necessitates consideration of various neoplastic entities such as NPC, metastases, clival chordoma, and non-neoplastic conditions including fibrous dysplasia, Paget’s disease, and granulomatous diseases [1].

Treatment of SBO primarily involves the administration of broad-spectrum intravenous antibiotics for a minimum duration of 3 months, with three main protocols commonly employed: aminoglycoside combined with a β-lactamase antibiotic, a third-generation cephalosporin such as Ceftazidime, or oral ciprofloxacin [2]. A recommended regimen includes intravenous administration of intravenous antibiotics for 4 to 6 weeks, followed by oral medication for 6 to 12 months, guided by clinical response [5].

Despite the effectiveness of prolonged antibiotic therapy, treatment failure can occur due to compromised tissue perfusion and oxygenation [2].

The impact of adjunctive surgical measures, such as surgical debridement and sequestrectomy, remains uncertain, with conflicting evidence regarding their effect on patient survival. However, surgical intervention can facilitate the removal of necrotic tissue, thereby potentially enhancing the penetration of antibiotics [5]. In specific cases, surgery may also be indicated to alleviate pressure on cranial nerves and manage complications such as abscesses and vascular issues [1, 5].

The role of hyperbaric oxygen therapy in the treatment of SBO is not well-defined, but may confer benefits such as improved tissue oxygenation, augmentation of the immune response against bacterial pathogens, and stimulation of angiogenesis [1, 5].

In conclusion, SBO represents a complex clinical entity necessitating a multidisciplinary approach for accurate diagnosis and optimal management. Awareness of predisposing factors, clinical features, diagnostic modalities, and treatment strategies is essential for successful patient outcomes.
